# The Persistence of the Diphtheria Bacillus

**Published:** 1902-10-04

**Authors:** 


### THE PERSISTENCE OF THE DIPHTHERIA BACILLUS.

Speaking at Manchester, Professor Sims Woodhead said that one of the most important points shown by the bacteriology of diphtheria was that the period during which it was necessary to isolate diphtheria patients was very much longer than had at one time been imagined. One could now understand, he said, how it was that fresh outbreaks of diphtheria had from time to time occurred after the disease had appeared to have died out. Referring to the cases which had been examined for the metropolitan Asylums Board he showed that the persistence of the bacilli for periods up to eight weeks was very common ; in fact the majority of the cases appeared to retain the bacilli in their throats for from two to nine weeks. After the ninth week the number undoubtedly fell off, but two cases came under observation which were found to have the throat infected with diphtheria bacilli for a longer period than 200 days, whilst 202 cases remained infective for a longer period than 100 days. Such details, he says, at once suggest the very important part that this persistence of the bacillus may play in determining the spread of the disease through old cases which are supposed to be no longer infective. Bearing this in mind and remembering that the main excuse for the expenditure of the rates in supporting isolation hospitals is that the community may be thereby protected from the further diffusion of infection, it is a matter of considerable interest and will, we think, be to some the cause of not inconsiderable surprise, to hear from Professor Woodhead that during the time that he was engaged examining cases for the Metropolitan Asylums Board nearly half of those examined in his laboratories were dismissed from hospital, although at the last examination diphtheria bacilli were still present. To the ordinary man it must often be difficult to reconcile the doings of the various sanitary authorities with which London is blessed, but seldom will he find a more striking example of the manner in which different public bodies are allowed to pull different ways and so to neutralise each other's action than that presented by the efforts made by certain local authorities to promote bacteriological diagnosis and to enforce the removal to hospital of cases shown to have diphtheria bacilli in their throats, whilst all the time the hospitals (which are under another and of course independent authority) are discharging patients wholesale who still carry about them these very bacilli, and are thus still capable of disseminating the disease !

### THE CAUSE OF SUMMER DIARRHŒA.

It has recently been stated that the specific germ responsible for the causation of infantile summer diarrhœa has been at last identified, and has been found to be the same as that which causes one form of dysentery, the Baccillus dysenteriæ of Shiga. From a statement in the *Medical Record*, authorised by Professer William H. Welch, it seems that as the result of an investigation of the causation of summer diarrhœa, made at the Thomas Wilson Sanatorium, near Baltimore, with the aid of a grant by the Rockfeller Institute of Medical Research, the Shiga bacillus was found in over forty cases of infantile summer diarrhœa. It was present in large numbers in acute cases, but was obtained with difficulty from mild and chronic cases. On the other hand, it was not found in the stools of healthy infants, nor of those affected with other diseases, many of whom were examined as controls. Confirmation of the relation between the bacilli and the disease is found in the agglutinative effect upon these bacilli which is exercised by the blood of the infants attacked, and the investigators believe that this bacillus is the cause of at least most cases of summer diarrhœa. It must be remembered, however, that summer diarrhœa has already been worked at by many observers and has been tentatively attributed to a considerable number of different micro-organisms. Such a suggestion as this, which means that a micro-organism capable of producing dysentery is as widely distributed as is infantile summer diarrhœa, is one which cannot be accepted until investigations on this particular point have been carried out over a much wider area, and especially in countries where dysentery is a rare disease.

